# A case of postoperative bullous allergic contact dermatitis caused by injection with lidocaine

**DOI:** 10.1111/cod.13297

**Published:** 2019-05-21

**Authors:** Angelique N. Voorberg, Marie L. A. Schuttelaar

**Affiliations:** ^1^ Department of Dermatology University of Groningen, University Medical Center Groningen Groningen The Netherlands

**Keywords:** allergic contact dermatitis, bullous contact dermatitis, case report, lidocaine, patch test

Injected local anaesthetics have been reported to cause delayed‐type reactions.^1^, ^2^, ^3^ A bullous type IV allergic reaction caused by lidocaine injected during a skin biopsy has been reported once.^4^

## CASE REPORT

**Figure 1:**
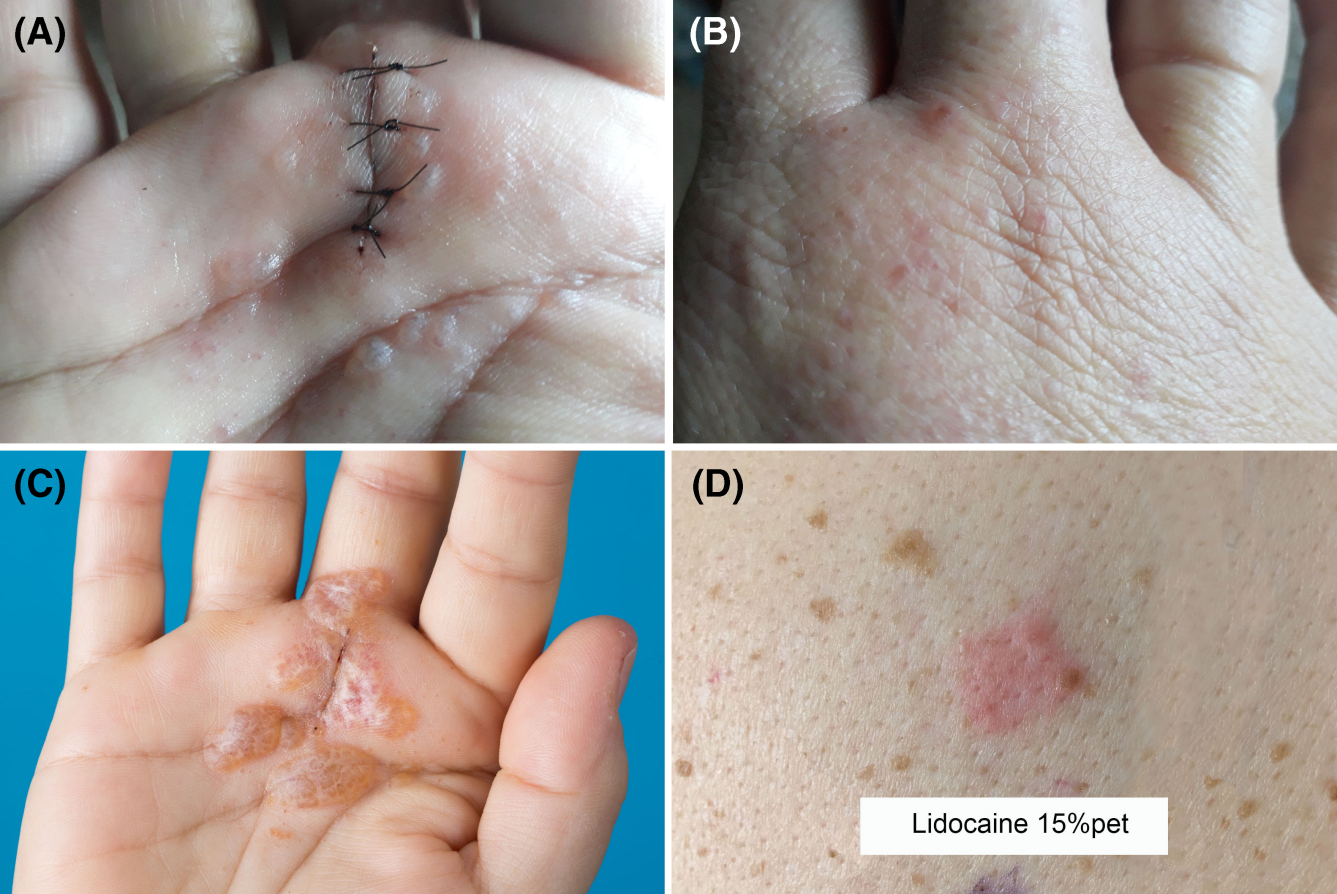
(**A,B**), Erythematous, vesicular and popular eruption at the lidocaine injection site. (**C**), Twelve days postoperatively, showing several bullae and multiple vesicles. (**D**), Patch test with lidocaine 15% pet. on day 3: ++

A 51‐year‐old woman underwent trigger finger surgery in which lidocaine 20 mg/mL with adrenaline 1:100 000 was injected subcutaneously as a local anaesthetic. Over the next 12 hours, she developed a pruritic erythematous, vesicular and papular eruption on her right palm and dorsum near the fourth metacarpal bone. Approximately 24 hours later, the vesicles developed into several painful bullae. Twelve days after the surgery, the bullae gradually decreased in severity (Figure 1A‐C).

Initially, the patient was referred to the Internal Medicine Allergology department because of a suspected postoperative allergic reaction to lidocaine. Intradermal testing with lidocaine 0.01 mg/mL (0.001%), 0.1 mg/mL (0.01%) and 1 mg/mL (0.1%) gave negative results after 15 minutes and 24 hours. Subsequently, the patient was referred to our Dermatology department and patch tested with our departmental extended European baseline series (TRUE Test panels 1 and 2 [SmartPractice Europe, Reinbek, Germany], supplemented with additional investigator‐loaded allergens), a local anaesthetics series (Chemotechnique Diagnostics, Vellinge, Sweden), lidocaine hydrochloride (HCl) 20 mg/mL (2%) with 1:10 0000 adrenaline “as is”, and lidocaine hydrochloride (HCl) 20 mg/mL (2%) “as is.” All investigator‐loaded allergens were tested in Van der Bend square chambers (Van der Bend, Brielle, The Netherlands), and all patch tests were attached to the back with Fixomull stretch (BSN Medical, Hamburg, Germany) for 2 days. Readings were performed on day (D) 3 and D7 according to the guidelines of the International Contact Dermatitis Research Group and the ESCD.^5^ The patient showed positive reactions to lidocaine 15% pet. (Figure 1D), lidocaine HCl 20 mg/mL (2%) with 1:10 0000 adrenaline, and lidocaine HCl 20 mg/mL (2%) “as is” (Table 1). No cross‐reactions with other local anaesthetics were observed. Additional patch testing with the steroid series was performed, because the patient showed a positive reaction to budesonide 0.1% pet. in the baseline series; positive reactions to triamcinolone acetonide 1% in ethanol, fluocinolone acetonide 1% eth., hydrocortisone acetate 1% eth. and methyl prednisolone 1% eth. were observed.

**Table 1:** Patch test results with the local anaesthetics series

Allergen	Concentration (%)	Vehicle	Day 3	Day 7
Lidocaine HCl	15	pet.	++	++
Lidocaine HCl (ampulla)	2	“as is”	++	++
Lidocaine HCl with adrenaline 1:100 000 (ampoule)	2	“as is”	+++	+++
Sodium metabisulfite	1	pet.	−	−
Mepivacaine HCl	2	pet.	−	−
Prilocaine HCl	5	pet.	−	−
Articaine HCl	5	pet.	−	−
Bupivacaine HCl	2	pet.	−	−
Ropivacaine HCl	1	pet.	−	−
Tetracaine HCl	5	pet.	−	−
Procaine HCl	2	pet.	−	−
Oxybuprocaine HCl	1	pet.	−	−

Abbreviations: HCl, hydrochloride.

## DISCUSSION

We present a patient with a bullous type IV allergic reaction to lidocaine after subcutaneous injections with lidocaine as a local anaesthetic during surgery. In the past, the patient had developed an eczematous reaction on her right wrist after surgery for carpal tunnel syndrome, without bullae, which was probably also attributable to lidocaine. As the patient had never experienced allergic reactions to disinfectants or dressing materials, it is unlikely that one of these was the culprits. The contact allergies to several steroids were most likely attributable to multiple corticosteroid injections for her trigger finger in the past.

Corbo et al reported that, in patients with a positive patch test reaction to lidocaine, both intradermal testing and subcutaneous testing should be performed to determine whether or not lidocaine could be used as a local anaesthetic in the future.^6^ In our patient, intradermal tests with lidocaine were read after 15 minutes and 24 hours. The test concentrations were low because higher concentrations can give irritant (false‐positive) wheal‐and‐flare reactions after 15 minutes.^1^ For detection of a delayed‐type reaction at 24 hours, the concentrations tested were probably too low. Subcutaneous testing with lidocaine was not performed, because it was very likely that lidocaine was the culprit allergen in this case.

## CONFLICTS OF INTEREST

The authors have no conflicts of interest to report.
